# Effectiveness and Safety of Rituximab for Refractory Myasthenia Gravis: A Systematic Review and Single-Arm Meta-Analysis

**DOI:** 10.3389/fneur.2021.736190

**Published:** 2021-10-13

**Authors:** Cong Zhao, Meng Pu, Dawei Chen, Jin Shi, Zhuyi Li, Jun Guo, Guangyun Zhang

**Affiliations:** ^1^Department of Neurology, Air Force Medical Center of PLA, Beijing, China; ^2^Department of Hepatobiliary Surgery, Air Force Medical Center of PLA, Beijing, China; ^3^Department of Neurology, Tangdu Hospital, Air Force Medical University, Xi'an, China

**Keywords:** myasthenia gravis, refractory, rituximab, effectiveness, meta-analysis

## Abstract

**Background and Objective:** Myasthenia gravis (MG) is an autoimmune neuromuscular disease. Nearly 10–30% of patients with MG are refractory to conventional therapy. Rituximab (RTX), a monoclonal antibody targeting CD20, is increasingly used in autoimmune disorders. We performed a systematic review and meta-analysis to evaluate the effectiveness and safety of RTX for refractory MG.

**Methods:** Studies published between January 1, 2000 and January 17, 2021 were searched in PubMed, EMBASE, Cochrane Library, and ClincalTrails.gov. Primary outcomes included proportion of patients achieving minimal manifestation status (MMS) or better and quantitative MG (QMG) score change from baseline. Secondary outcomes were glucocorticoids (GC) doses change from baseline and proportion of patients discontinuing oral immunosuppressants.

**Results:** A total of 24 studies involving 417 patients were included in the meta-analysis. An overall 64% (95% confidence interval, 49–77%) of patients achieved MMS or better. The estimated reduction of QMG score was 1.55 (95% confidence interval, 0.88–2.22). The mean reduction of GC doses was 1.46 (95% confidence interval, 1.10–1.82). The proportion of patients discontinuing oral immunosuppressants was 81% (95% confidence interval, 66–93%). Subgroup analyses showed that the proportion of patients achieving MMS or better and discontinuing oral immunosuppressants was higher in MuSK-MG group than those in AChR-MG group. Improvement was more pronounced in patients with mild to moderate MG compared to those with severe MG. Moreover, the efficacy appeared to be independent of the dose of RTX. 19.6% of patients experienced adverse events, most of which were mild to moderate. Only one patient developed progressive multifocal leukoencephalopathy.

**Conclusions:** RTX can alleviate the symptom of weakness, decrease QMG score and reduce the doses of steroids and non-steroid immunosuppressive agents in refractory MG. It is well-tolerated with few severe adverse events. Randomized controlled trials are urgently needed to study the efficacy of RTX in treating refractory MG and to identify the characteristics of patients who might respond well to RTX.

## Introduction

Myasthenia gravis (MG) is an acquired autoimmune disease of the neuromuscular junction (NMJ) characterized by partial or systemic skeletal muscle weakness and fatigability typically worsening after activity (1). It is caused by autoantibodies that target the functional molecules at the postsynaptic membrane of the NMJ. The estimated prevalence of MG is 150–250 per 1,000,000 with an annual incidence of 8–10 per 1,000,000 (2). Nearly 80–90% patients with MG present with antibodies (Ab) against acetylcholine receptor (AChR). 1–10% of patients have antibodies against muscle-specific tyrosine kinase (MuSK). The remaining patients may demonstrate antibodies against lipoprotein-related protein 4 (LRP4) or agrin in the postsynaptic membrane at NMJ (3).

The management of MG aims to improve the symptoms of muscle weakness and quality of life while minimizing the drug side effects (4). Current therapies in MG consists of acetylcholinesterase (AChE) inhibitors, conventional immunosuppressive treatments, short-term fast-acting therapy such as plasma exchange and intravenous immunoglobulin (IVIg) and thymectomy (4). Most patients experience significant relief with AChE inhibitors and traditional oral immunosuppressants. However, to achieve satisfactory therapeutic effect, patients usually require long-term or lifelong immunosuppressive therapy. Moreover, 10–30% of patients are refractory to conventional immunosuppression (3, 5, 6). They have persistent symptoms or frequent relapses, frequently require PE or IVIg therapy, or are intolerable to the side effects of immunosuppressive agents. Therefore, it is of great significance to find out effective and safe treatment options for refractory MG.

Rituximab (RTX) is a chimeric mouse/human monoclonal antibody which specifically deplete CD20 positive B lymphocytes (7). It was approved as therapy for non-Hodgkin B-cell lymphoma (8), as well as some autoimmune diseases such as rheumatoid arthritis (9) and systemic lupus erythematosus (10). In the past two decades, growing evidence have shown that patients with MG may benefit from RTX therapy (11). A previous meta-analysis showed that patients with MG responded well to RTX treatment, regardless of the serotypes of MG (12). However, this meta-analysis only assessed the response rate of RTX therapy. Recently, some new studies have emerged, and evaluated a variety of other outcome indicators such as post intervention status (PIS) defined by the Myasthenia Gravis Foundation of America (MGFA) (13), the dosage of prednisone, quantitative MG (QMG) score. Data about the changes of these outcomes in patients with AChR-MG and MuSK-MG after RTX therapy are still lacking. Therefore, we performed an updated meta-analysis to evaluate the effectiveness and safety of RTX therapy for the treatment of refractory MG, which might allow more extensive using of RTX in treating refractory MG.

## Methods

### Study Selection and Data Extraction

Our research was conducted according to the Preferred Reporting Items for Systematic Reviews and Meta-Analysis (PRISMA) guideline (14). The review protocol was registered on PROSPERO (registration number: CRD42021202634). Two reviewers (Cong Zhao and Meng Pu) independently searched PubMed, EMBASE, Cochrane Library, and ClincalTrails.gov for studies investigated the effectiveness and safety of rituximab treating MG. Since rituximab was approved for marketing in the late 1990s, the searching was limited to the period between January 1, 2000 and January 17, 2021. A combination of searching keywords and medical subject headings (MeSH) were used as follows: (“myasthenia gravis” OR “myastheni^*^”) AND (“Rituximab” OR “IDEC-C2B8” OR “anti-CD20”). The detailed specific searching strategy for each database was supplied in Supplementary Material A.

### Inclusion and Exclusion Criteria

#### Participants

We included patients with refractory MG, which was defined as insufficient response to corticosteroids and oral immunosuppressants despite adequate doses and duration, or intolerable adverse effects from conventional immunosuppressive therapy (4).

#### Interventions

We included studies with a RTX arm assessing the effectiveness and safety of RTX to treat patients with refractory MG.

#### Outcome Measures

Two primary effectiveness outcomes were included: (1) the proportion of patients achieving minimal manifestation status (MMS) or better. MMS is defined as that the patient has no symptoms or functional limitations from MG but has weakness on examination of some muscles (13); (2) quantitative MG (QMG) score (13) change after RTX therapy. The secondary outcomes were the proportion of patients discontinuing oral immunosuppressants and glucocorticoids (GC) doses change after RTX therapy. The safety outcomes included infusion reactions, allergic reactions, infections, hematological disorder and proportion of deaths.

#### Studies

We searched for randomized controlled trials (RCTs), observational studies and single-arm researches limited to human studies published in English. We excluded small case series with fewer than three patients. Studies lacking clinical data were also excluded from meta-analysis. Two reviewers (Cong Zhao and Dawei Chen) read the full text of the relevant studies to evaluate the appropriateness for their inclusion in the meta-analysis.

### Data Extraction

For each study, author's name, study design, publication year, country, patients' characteristics, RTX regimens, and outcome measures were extracted. The following patients' characteristics were retrieved when available: sample size, the presence of MG-related autoantibody, proportion of female, age at initiation of RTX therapy, disease duration, follow-up duration, therapy prior to RTX. Data were extracted by two reviewers (Cong Zhao and Meng Pu) independently from each study with a data extraction form and verified by a third reviewer (Dawei Chen). Consensus was reached through discussion when any contradiction appeared.

### Statistical Analysis

The meta-analysis was performed with R software 4.0.5 (The R Foundation for Statistical Computing, Vienna, Austria). Due to the presence of trials with zero events, the data of binary variables were firstly converted into double arcsine-transformed proportions and then merged using DerSimonian-Laird method with random effects model. Standard mean difference (SMD) with 95% confidence intervals (CI) were calculated using a random effects model to quantify the changes of continuous variables before and after RTX treatment. Statistical difference was set as *P* < 0.05. Representative forest plots conveyed an overview of the results and details of the included studies and combined effects. The between-study heterogeneity was analyzed by chi-square test with *I*^2^ statistics. Publication bias were evaluated using funnel plot and Egger test. Sensitivity analyses were conducted to estimate and validate the impact of each study on the pooled results. If sufficient subgroup data were available, a further subgroup analyses were carried out to evaluate the outcome measures among different MG subtypes.

## Results

### Study Characteristics

**Figure 1:**
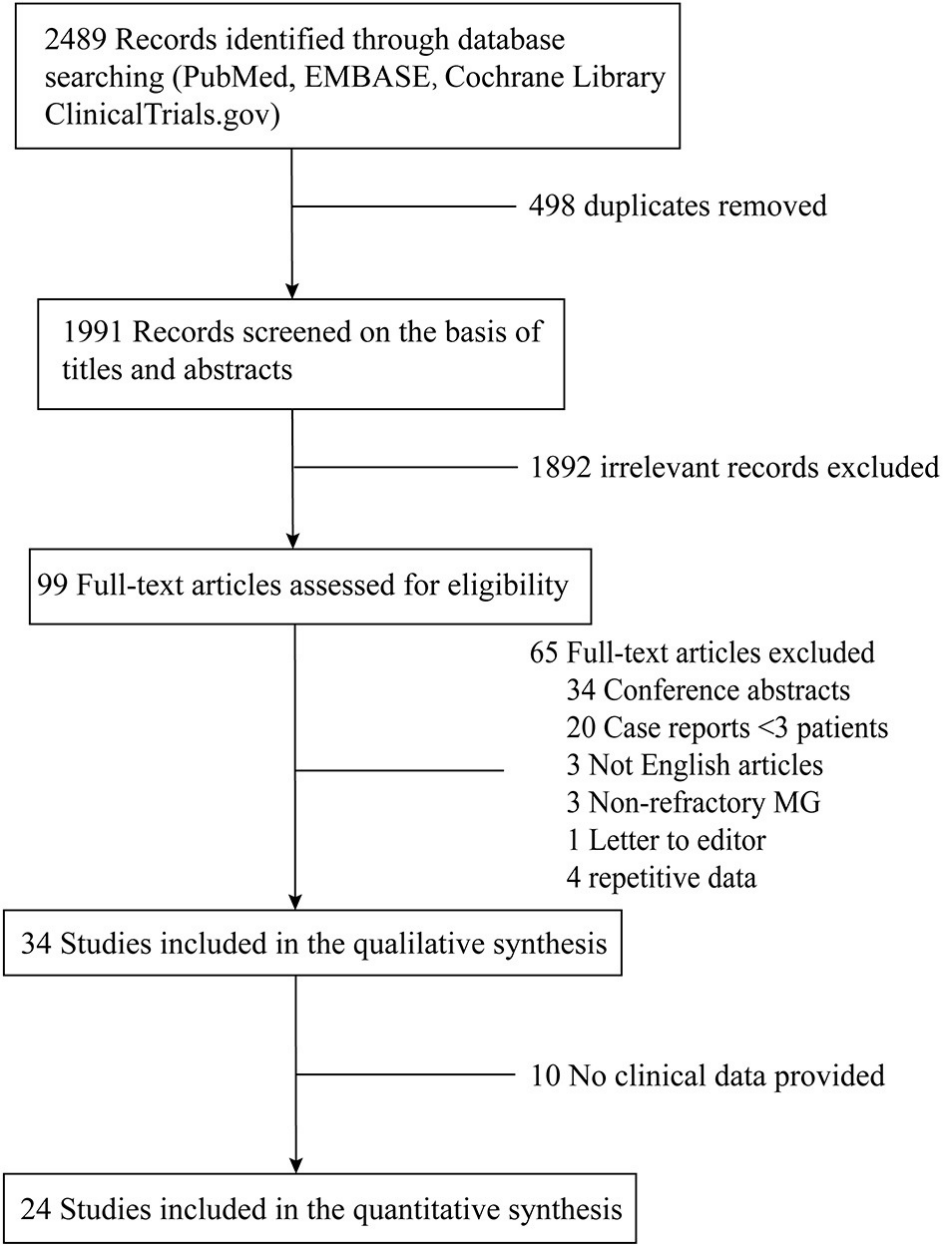
Flow chart of study selection algorithm according to PRISMA guidelines.

A flow-chart of the search strategy and study selection was shown in Figure 1. There were 99 relevant studies identified through thoroughly database searching after duplicates removing and title/abstract screening. The data from two studies by Jing et al. (15, 16) were pooled together because the authors described the same patients cohort at different time points, so were the data from the studies by Anderson et al. (17, 18) and Topakian et al. (19–21). Finally, 24 studies (16, 18, 21–42) were included in the meta-analysis. All the studies were single-arm observational studies except for one RCT by Hehir et al. comparing patients with MuSK-MG treated with RTX to those not treated with RTX (32).

A total of 417 patients (112 male and 305 female) with refractory MG were treated with RTX in these 24 studies, of whom 242 were AChR-IgG positive, 155 were MuSK-IgG positive, and 20 were defined as “double seronegative” (DN). The mean age of all patients at onset of RTX therapy was 43.8 years (not specified in five studies). The mean disease duration at onset of RTX therapy was 96.4 months (not specified in six studies). Table 1 summarized the demographic and clinical characteristics of the patients included in the meta-analysis.

**Table 1:** Baseline characteristics of studies included in the meta-analysis.

**References**	**Country**	**Sample size (AChR/Musk/ DN)**	**Female, No. (%)**	**Age at initiation of RTX, mean (SD), years**	**Disease duration before RTX, mean (SD), months**	**Follow-up after RTX, mean (SD), months**	**RTX regimen**
Litchman et al. (22)	USA	33(17/16/0)	24(73)	35.9(15.6)	53.2(80.8)	62.1(31.8)	Four weekly infusions of 375 mg/m^2^. One cycle was defined as 1 infusion per week for 4 consecutive weeks. The interval between cycles was 6 months.
Marino et al. (23)	Italy	9(0/9/0)	8(89)	50.4(12.8)	132(72.5)	51.8(38.2)	Four weekly infusions of 375 mg/m^2^, plus a single dose of 375 mg/m^2^ after 3 months.
Lu et al. (24)	China	12(12/0/0)	10(83)	30.6(29.6)	59.6(37.7)	18	600 mg rituximab intravenously every 6 months for three infusions at 0, 6, and 12 months.
Sahai et al. (25)	USA	7(7/0/0)	5(71)	NA	48.3(65.6)	4.6	1 g of RTX repeated after 2 weeks
Roda et al. (28)	USA	27(10/13/4)	22(81)	AchR 49(26~80)* MUSK 32(20~62)* DN 56(26~61)*	NA	NA	375 mg/m^2^ weekly for 4 consecutive weeks or rarely 1,000 mg at weeks 1 and 3. Retreatment was based on clinical course.
Choi et al. (26)	Korea	17(9/6/2)	11(65)	49.8(17.1)	148.8(102.0)	24.5(11.3)	375 mg/m^2^ twice with a 2-week interval followed by additional single infusions (375 mg/m^2^ once). Retreatment was according to clinical situation or B cells frequency.
Topakian et al. (21)	Austria	56(39/14/3)	34(61)	47.5(33~71)*	48(15.63~129.6)*	20(10~53.5)*	47 patients had induction therapy consisting of two RTX infusions within 2 weeks at a dose of 2 × 375 mg/m^2^ (*n* = 17), 2 × 500 mg (*n* = 15) or 2 × 1,000 mg (*n* = 15). Other protocols were used in nine patients.
Jing et al. (16)	China	15(14/1/0)	14(93)	34.4(13.1)	57.3(32.8)	6	100 mg on day1 and 500 mg on day2. A repeat cycle of 600 mg every 6 months was given according to clinical status and patient's preference
Singh et al. (27)	India	8(6/2/0)	7(88)	38.1(12.0)	154.5(90.8)	116.6(97.2)	Four weekly infusions of 375 mg/m^2^. One cycle was defined as 1 infusion per week for 4 consecutive weeks. Repeat cycle was planned at 6 month interval if required.
Beecher et al. (18)	Canada	22(10/9/3)	10(43)	49.4(13.4)	51.4(53.7)	28.8(19.0)	Induction regimen: 375 mg/m^2^ were given once weekly for 4 weeks, and once every 4 weeks for 2 additional infusions or 750 mg/m^2^ were given twice, with 2 weeks between infusions. Maintenance regimen: 2 doses of 750 mg/m^2^ (up to a maximum of 1 g per dose), with 2 weeks between infusions
Landon-Cardinal et al. (29)	France	11(11/0/0)	8(73)	41.5(12.3)	174(90)	18	Two infusions of 1 g of RTX separated by 2 weeks' intervals, followed by 1 g infusion 6 months after the day 14 injection.
Cortes-Vicente et al. (30)	Spain	25(0/25/0)	24(96)	51.4(15.8)	NA	60(39.6)	One of the three following protocols: (1) 375 mg/m^2^ every week for 4 consecutive weeks and then monthly for the next 2 months; (2) two 1 g doses separated by 2 weeks; (3) 375 mg/m^2^ every week for 4 consecutive weeks. Re-infusions were administered if patients relapsed.
Afanasiev et al. (31)	France	28(21/3/4)	16(57)	50.6(12.0)	135.6 (6–312)*	27.2(16.6)	Induction regimen: 1,000 mg on day and 15 or 375 mg/m^2^ weekly for 4 weeks. Maintenance regimen: 1,000 mg or 375 mg/m^2^ infusion, with a 6 months periodicity.
Hehir et al. (32)	USA	24(0/24/0)	21(88)	NA	NA	45(6~116)*	The initial dose of rituximab in all patients was 375 mg/m^2^ weekly for 4 weeks. Thirteen patients were re-treated with 375 mg/m^2^ weekly for 4 weeks. The other 2 patients were re-treated with 1,000 mg weekly for 2 weeks.
Peres et al. (33)	Portugal	6(4/0/2)	5(83)	62.0(16.0)	129.6(153.6)	39(11~67)*	2 infusions of 1,000 mg given 15 days apart. Retreatment is decided by experts based on disease activity, CD19 lymphocyte plasma count and serum immunoglobulin levels, with a minimum interval between infusions of 4 months.
Robeson et al. (34)	USA	16(16/0/0)	10(63)	40.6(16.8)	35.7(24.6)	56.1(20.1)	Four weekly infusions of 375 mg/m^2^. One cycle was defined as 1 infusion per week for 4 consecutive weeks. The interval between cycles was 6 months.
Sun et al. (35)	China	22(15/7/0)	15(68)	42.3(11.5)	72(36.2)	17(3.66)	Four weekly infusions of 375 mg/m^2^. Reinfusion of rituximab with a single dose of 375 mg/m^2^ was initiated when circulating CD19 B cells exceeded 1%
Díaz-Manera et al. (36)	Spain	17(11/6/0)	15(88)	44.3	MUSK 128 AchR 120	31	Four weekly infusions of 375 mg/m^2^ and then monthly for the next 2 months. Repeat rituximab infusions were administered when myasthenic symptoms reappeared and interfered with daily life activities.
Collongues et al. (37)	France	13(8/3/2)	8(62)	NA	138.9(142.9)	26(13)	Eleven patients received 375 mg/m^2^ weekly for 4 consecutive weeks (induction stage), and subsequently 375 mg/m^2^ every 3 months. Two patients received 2 infusions of 1 g with 2 weeks apart (induction stage), and subsequently 1 g, as required if symptoms worsened.
Nowak et al. (38)	USA	14(6/8/0)	11(79)	46.0(13.5)	NA	12	Four weekly infusions of 375 mg/m^2^. One cycle was defined as 1 infusion per week for 4 consecutive weeks. The interval between cycles was 6 months.
Blum et al. (39)	Australia	14(11/3/0)	9(64)	51.1(18.4)	127.7(152.0)	14.4(11.3)	500 mg twice, 2 weeks apart. Retreatment was initiated if B cells count over 1% on two separate occasions, together with clinical signs of relapsing disease,
Maddison et al. (40)	UK	10(7/3/0)	10(100)	32.7(12.2)	121.2(110.3)	12~48	375 mg/m^2^ weekly for 4 consecutive weeks. Additional once-monthly infusions were administrated in three patients.
Lindberg et al. (41)	Sweden	5(5/0/0)	3(60)	24.4(9.5)	256.8(178.8)	NA	Four weekly infusions of 375 mg/m^2^. In some patients, RTX re-treatment was given as two infusions of 1000 mg 2 weeks apart.
Illa et al. (42)	Spain	6(3/3/0)	5(83)	48.3(16.1)	105.6(84.0)	9	Four weekly infusions of 375 mg/m^2^ and then monthly for the next 2 months.

NA, not available; *median (range).

The dose regimen of RTX therapy varied among studies. 284 patients received routine induction doses of RTX, namely 375 mg/m^2^ weekly for 4 consecutive weeks or 1 g twice within 2 weeks apart. 60 patients received low induction doses of RTX, including 27 patients received 600 mg RTX, 16 patients received 1 g RTX, and 17 patients received 375 mg/m^2^ twice with a 2-week interval. Reinfusions were usually decided according to clinical manifestations or the frequency of B cells.

### Effectiveness Outcome Measures

#### Proportion of Patients Achieving MMS or Better

**Figure 2:**
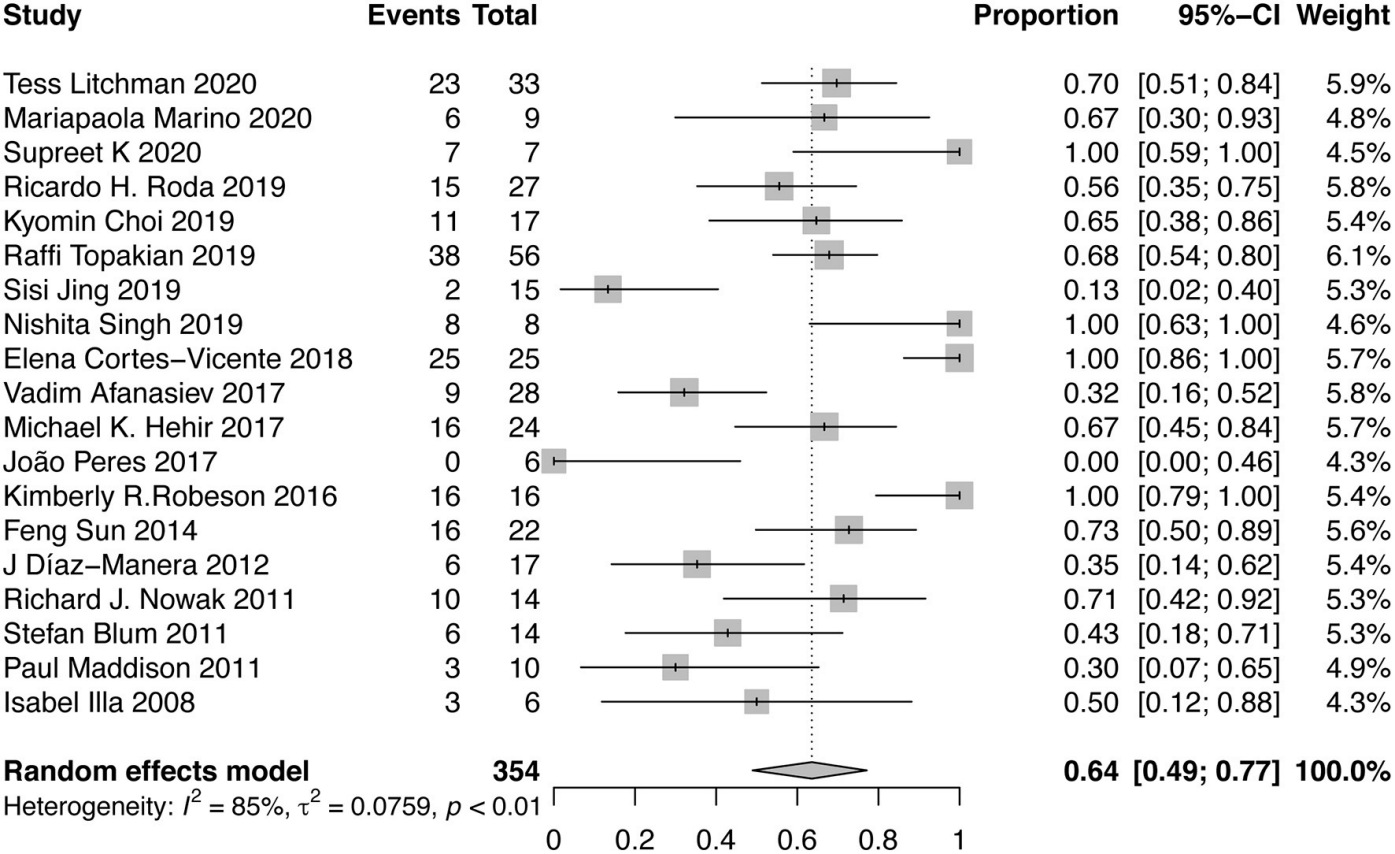
Forest plot showing the mean effect size and 95% CI for the proportion of patients achieving MMS or better.

The achievement of MMS or better was reported in 19 studies. A forest plot was shown in Figure 2. Overall, there were 64% (95% CI, 49–77%) of patients achieving MMS or better. Abundant between-study heterogeneity was detected (*I*^2^ = 85%). Sensitivity analysis showed a relatively stable result (Supplementary Material B Figure 1). The funnel plot was symmetrical (Supplementary Material B Figure 2), and the result of Egger test suggested no publication bias (*P* = 0.6552). Subgroup analysis was performed among the studies which provided sufficient subgroup data to assess the effect of MG subtype, severity, and RTX dose on the results. There was 51% (95%CI, 31–70%; *I*^2^ = 81%) of patients achieving MMS or better in AChR-MG group, 79% (95%CI, 64–92%; *I*^2^ = 64%) in MuSK-MG group and 40% (95%CI, 9–74%; *I*^2^ = 22%) in DN-MG group. 72% (95%CI, 44~95%; *I*^2^ = 83%) of patients with mild to moderate MG (MGFA clinical classification I~III) achieved MMS or better, and so did 45% (95%CI, 30~60%; *I*^2^ = 24%) of patients with severe MG (MGFA clinical classification IV~V). There was 67% (95%CI, 51~82%) of patients receiving routine dose of RTX and 48% (95%CI, 16~81%) of patients receiving low dose of RTX achieved MMS or better, respectively (Table 2).

**Table 2:** Subgroup analysis for outcome measures in patients with MG.

**Outcome measures**	**Subgroups**	**Effect size**	**95%CI**	***I* ^**2**^**
Proportion of patients achieving MMS or better	AChR-MG	0.51	0.31~0.70	81%
	MuSK-MG	0.79	0.64~0.92	64%
	DN-MG	0.40	0.09~0.74	22%
	MGFA I~III	0.72	0.44~0.95	83%
	MGFA IV~V	0.45	0.30~0.60	24%
	Routine dose	0.67	0.51~0.82	86%
	Low dose	0.48	0.16~0.81	75%
The doses of GC	AChR-MG	−1.41	−0.78~-2.03	67%
	MuSK-MG	−1.38	−1.05~-1.71	0%
	DN-MG	−0.33	−1.67~-1.01	0%
Proportion of patients discontinuing oral immunosuppressants	AChR-MG	0.69	0.39~0.93	74%
	MuSK-MG	0.97	0.87~1.00	0%
	DN-MG	0.91	0.44~1.00	29%

#### QMG Score

**Figure 3:**
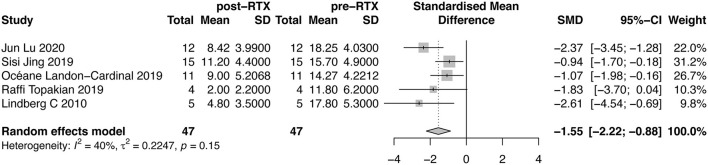
Forest plot showing the mean effect size and 95% CI for the reduction of QMG score.

Five studies evaluated the changes of QMG score. A forest plot with SMD of QMG score before and after RTX therapy was shown in Figure 3. The mean reduction of QMG score was 1.55 (95%CI, 0.88–2.22). We did not detect publication bias through Egger test (*P* = 0.4449, Supplementary Material B Figure 3). The between-study heterogeneity was determined by *I*^2^ test (*I*^2^ = 40%). Due to insufficient subgroup data, we did not conduct subgroup analysis on this outcome measure.

#### The Doses of GC

**Figure 4:**
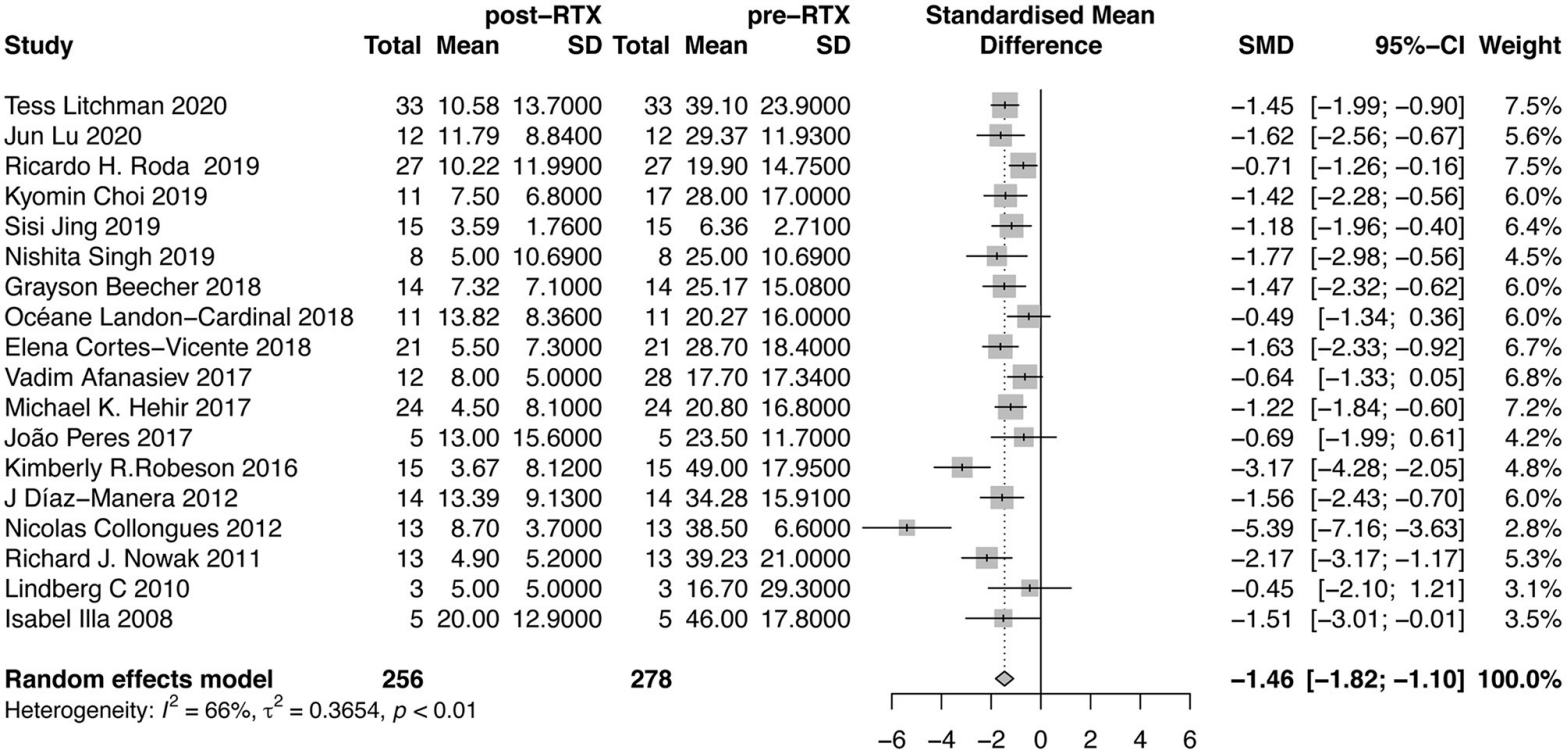
Forest plot showing the mean effect size and 95% CI for the reduction of GC doses.

**Figure 5:**
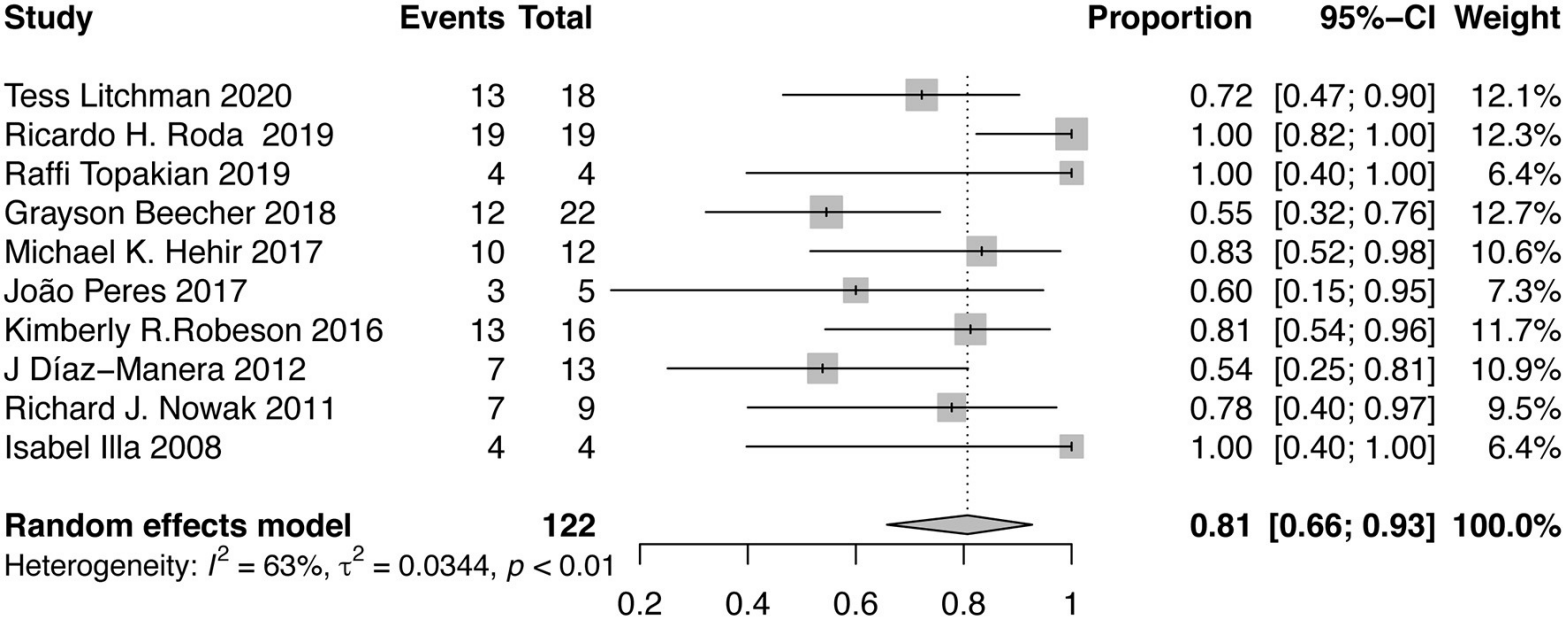
Forest plot showing the mean effect size and 95% CI for the proportion of patients discontinuing oral immunosuppressants.

The doses of GC were reported in 18 studies (Figure 4). Our meta-analysis suggested that mean reduction of GC dose after RTX therapy was 1.46 (95%CI, 1.10–1.82). The between-study heterogeneity was high (*I*^2^ = 66%). Sensitivity analysis revealed that the result was relatively stable after omitting each study in turn (Supplementary Material B Figure 4). No publication bias was detected (*P* = 0.0559, Supplementary Material B Figure 5). In further subgroup analysis, the average dose reduction of GC was 1.41 (95%CI, 0.78–2.03; *I*^2^ = 67%) in AChR-MG group, 1.38 (95%CI, 1.05–1.71; *I*^2^ = 0%) in MuSK-MG group and 0.33 (95%CI, 1.01–1.67; *I*^2^ = 0%) in DN-MG group, as shown in Table 2.

#### Proportion of Patients Discontinuing Oral Immunosuppressants

Ten studies that reported the proportion of patients discontinuing oral immunosuppressants after RTX therapy were included in the meta-analysis. After RTX treatment, 81% (95%CI, 66–93%; *I*^2^ = 63%) of the patients stopped taking oral immunosuppressants (Figure 5). Sensitivity analysis indicated that the result was stable (Supplementary Material B Figure 6). No publication bias was detected (*P* = 0.6529, Supplementary Material B Figure 7). The proportion of AChR-MG patients discontinuing oral immunosuppressants was 69% (95%CI, 39–93%), while in MuSK-MG and DN-MG patients, the ratio was 97% (95%CI, 87–100%) and 91% (95%CI, 44–100%), respectively (Table 2).

### Safety

Adverse effects (AEs) were studied in 363 patients and 71 AEs (19.6%) were recorded. Specifically, 39 patients (10.7%) experienced infusion reactions, 21 patients (5.8%) developed infections and 7 patients (1.9%) developed hematological disorders. Other uncommon AEs included mental disorder (0.2%), alopecia areata (0.2%), paroxysmal atrial fibrillation (0.2%). Only one patient (0.2%) was histologically diagnosed with progressive multifocal leukoencephalopathy (PML).

## Discussion

RTX was firstly reported to be effective in treating MG in 2000 (44). In the past 20 years, a considerable number of studies reported successful using RTX as a second-line therapy for refractory MG. A previous meta-analysis showed that 83.9% of patients improved PIS after RTX treatment. Here, in our updated meta-analysis, we re-evaluated the effectiveness of RTX from more dimensions. Firstly, we found that 64% of patients achieved MMS or better in PIS following RTX therapy, which is the goal for the treatment of MG (4). Secondly, RTX significantly decreased the QMG score by 1.55 and the GC doses by 1.46. Moreover, 81% patients stopped taking oral immunosuppressants at last visit. In conclusion, RTX could effectively alleviate the symptoms of MG, decrease QMG score and reduce the doses of steroids and non-steroid immunosuppressive agents.

Some studies suggested that the benefits of RTX might depend on the serotype of MG. Patients with MuSK-MG maintained a sustained disease control and withdraw immunosuppressants more quickly compared to their AChR-MG counterparts (18, 21, 22, 36). Our analysis revealed that the proportion of achieving MMS or better and the proportion of stopping oral immunosuppressants in MuSK-MG was higher than those in AChR-MG, but the differences between subgroups were not statistically significant because of the overlapping of the 95% CI. The previous meta-analysis showed that PIS was improved in 80.4% of patients with AChR-MG and 88.8% of patients with MuSK-MG (12). While, in our study, 51% of AChR-MG patients and 79% of MuSK-MG patients achieved MMS or better. The difference between the two subgroups enlarged when the outcome measures become more stringent. Together, these results suggested that MuSK-MG might be a little more sensitive to RTX than AChR-MG and patients with MuSK-MG might achieve the treatment goal more easily compared to AChR-MG patients. Different responses to RTX therapy may be accounted by the pathogenesis discrepancy of AChR-MG and MuSK-MG. Most anti-MuSK Abs are IgG4 and secreted by short-lived plasma cells, the precursors of which are CD20 positive B cells. Depleting CD20 positive B cells by RTX results in dramatical reductions of the population of short-lived plasma cells and the titer of anti-MuSK Abs (45). Nevertheless, anti-AChR Abs, belonging to IgG1 and IgG3 subclasses, are mostly secreted by long-lived plasma cells. Their population size is self-sustaining and not affected by B-cell depletion (43). However, not all outcome measures differed between AChR-MG and MuSK-MG. The two groups were very close in the dose change of GC after RTX therapy. This may be due to the fact that patients with MuSK-MG was steroids-dependent, thus counteracting the superiority of MuSK-MG over AChR-MG during RTX therapy. Different response to RTX might also be related to disease duration and severity. A retrospective study revealed that time to remission after RTX treatment was shorter in newly onset patients compared with patients with refractory MG (46). Therefore, more studies should be carried out to identify the characteristics of patients who benefit significantly from RTX treatment.

In order to clarify whether there were differences in the efficacy of RTX in patients with different disease severity, subgroup was performed. In fact, the proportion of patients achieving MMS or better was higher in mild to moderate patients than severe patients, but the difference was not statistically significant because 95%CI overlapped. This suggested that RTX therapy could be initiated as soon as possible in refractory MG instead of waiting until the disease worsened. The dose of RTX may be another important factor affecting RTX efficacy. Subgroup analysis showed there was no difference in proportion of patients achieving MMS or better between the low dose group and the routine dose group. Our result was similar to a previous meta-analysis in AChR-MG (47). These two studies preliminarily indicated that low dose RTX was as effective as routine dose. Furthermore, some studies suggested that low dose RTX was less likely to have serious side effects (48). Due to the limited number of patients using the low dose regimen, these conclusions were not necessarily reliable. Further clinical studies should be conducted to evaluate the long-term efficacy and safety of low dose RTX in treating refractory MG.

RTX was well-tolerated in patients with MG. Even though most of the patients were treated with GC and immunosuppressants at the same time, adverse effects were only observed in 19.6% of (71 of 363) patients. This is similar to adverse effects rates of RTX in treating other autoimmune diseases (48–50). The most common adverse effects were infusion reactions, which could be easily prevented by antihistamines and steroids. Several studies reported that infusion reactions became less frequently when RTX was reinjected. 5.8% of patients had treatable infections such as pneumonia, herpes zoster, viral gastroenteritis and cholecystitis. Only one patient (0.2%) was diagnosed with PML, which is a fatal infection of brain caused by John Cunningham virus and usually infects immune-compromised patients. This patient received a total dose of 10 g RTX concomitant with prednisone, azathioprine and mycophenolate mofetil (31). Despite the rarity of PML, clinicians should take it into consideration when patients have manifestation of encephalopathy.

There were some limitations in our study. Firstly, most of the studies included in the meta-analysis were observational studies, which might overestimate the effectiveness of treatments compared with controlled trails. Secondly, we could not compare the efficacy of RTX with other drug since most of the included studies were single-arm. Thirdly, the number of patients in each study was relatively small. In subgroup analysis, the number of cases in some studies was no more than 5, which resulted in great randomness of research results. Finally, the heterogeneity between studies was remarkable. There were many reasons for the high heterogeneity. MG is a rare disease with high heterogeneity. Moreover, the RTX regimen, follow up duration and baseline characteristics of patients differed among studies. We could not perform meta-regression because some information was inaccessible in studies. Therefore, a large sample randomized controlled trial is necessary to evaluate the effectiveness and safety of RTX in treating refractory MG.

## Conclusions

In conclusion, this systemic review and meta-analysis suggested that RTX therapy could improve the PIS of a considerable number of patients with refractory MG to reach MMS or better with a good safety profile. It also exhibited a steroid-sparing effect. Furthermore, RTX reduced QMG scores and the use of conventional oral immunosuppressants. The efficacy was related to the patient's serotype and disease severity, but not to the doses of RTX. It is necessary to conduct RCTs to compare the efficacy and safety between RTX and other treatments, and to explore the characteristics of patients who are sensitive to RTX therapy.

## Data Availability Statement

The original contributions presented in the study are included in the article/Supplementary Material, further inquiries can be directed to the corresponding authors.

## Author Contributions

CZ, JG, and GZ conceived the study. CZ and MP did the literature review. CZ, MP, and DC organized the database and performed the statistical analyses. CZ wrote the primary manuscript. JS and ZL critically revised the manuscript. All authors read and approved the final version of the manuscript.

## Funding

This study was supported by the National Natural Science Foundation of China (Grant No. 81901226).

## Conflict of Interest

The authors declare that the research was conducted in the absence of any commercial or financial relationships that could be construed as a potential conflict of interest.

## Publisher's Note

All claims expressed in this article are solely those of the authors and do not necessarily represent those of their affiliated organizations, or those of the publisher, the editors and the reviewers. Any product that may be evaluated in this article, or claim that may be made by its manufacturer, is not guaranteed or endorsed by the publisher.
