# Supplementary material for: Effectiveness and Safety of Rituximab for Refractory Myasthenia Gravis: A Systematic Review and Single-Arm Meta-Analysis
Source: Front Neurol. 2021 Oct 13;12:736190. doi: 10.3389/fneur.2021.736190 (PMC8548630; doi:10.3389/fneur.2021.736190)
Supplement: Supplementary file 1 [file Data_Sheet_1.docx]

**Supplementary Material A**

Searching strategy for each database

PubMed Search Strategy

#1 "Myasthenia Gravis"[Mesh]

#2 myastheni*[Title/Abstract]

#3 #1 OR #2

#4 "Rituximab"[Mesh]

#5 "Antibodies, Monoclonal"[Mesh]

#6 "Antigens, CD20"[Mesh]

#7 ritux*[Title/Abstract]

#8 "IDEC C2B8"[Title/Abstract]

#9 IDEC-C2B8[Title/Abstract]

#10 Mabth*[Title/Abstract]

#11 "CD20 antibody"[Title/Abstract]

#12 anti-CD20[Title/Abstract]

#13 monocl*[Title/Abstract] AND antibo*[Title/Abstract]

#14 OR/4-13

#15 3 AND 14

EMBASE Search Strategy

#1 'myasthenia gravis'/exp

#2 myastheni$:ab,ti

#3 1 OR 2

#4 'rituximab'/exp

#5 'monoclonal antibody'/exp

#6 'cd20 antigen'/exp

#7 ritux*:ab,ti

#8 'idec c2b8':ab,ti

#9 mabth*:ab,ti

#10 'cd20 antibody'/exp

#11 'anti cd20':ab,ti

#12 monocl*:ab,ti AND antibo*:ab,ti

#13 OR/4-12

#14 3 AND 13 AND [2000-2021]/py

The Cochrane Library Search Strategy

#1 "Myasthenia Gravis"[Mesh]

#2 myastheni*[Title/Abstract]

#3 1 OR 2

#4 "Rituximab"[Mesh]

#5 "Rituxan"[ Title/Abstract]

#6 "mabthera"[ Title/Abstract]

#7 OR/4-6

#8 3 AND 7

ClinicalTrials.gov Search Strategy

(Rituximab) AND (Myasthenia Gravis)
