# Supplementary material for: Effectiveness and Safety of Rituximab for Refractory Myasthenia Gravis: A Systematic Review and Single-Arm Meta-Analysis
Source: Front Neurol. 2021 Oct 13;12:736190. doi: 10.3389/fneur.2021.736190 (PMC8548630; doi:10.3389/fneur.2021.736190)
Supplement: Supplementary file 2 [file Data_Sheet_2.docx]

**Supplementary Material B**


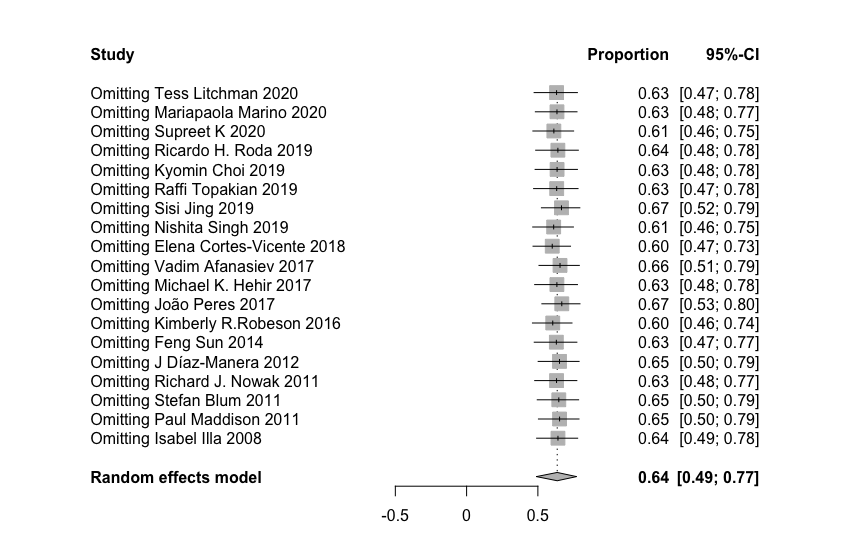
Supplementary material B Figure 1. Sensitivity analysis for the proportion of patients achieving MMS or better


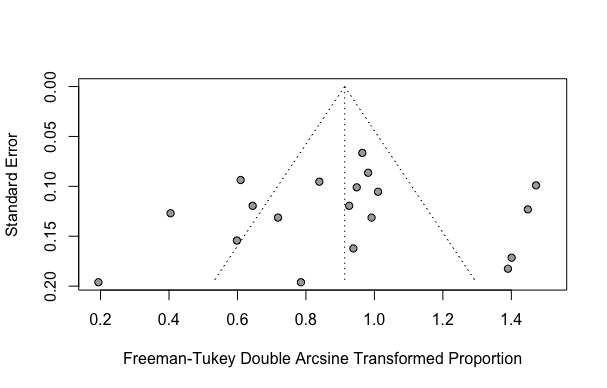


Supplementary material B Figure 2. The funnel plot of for the proportion of patients achieving MMS or better


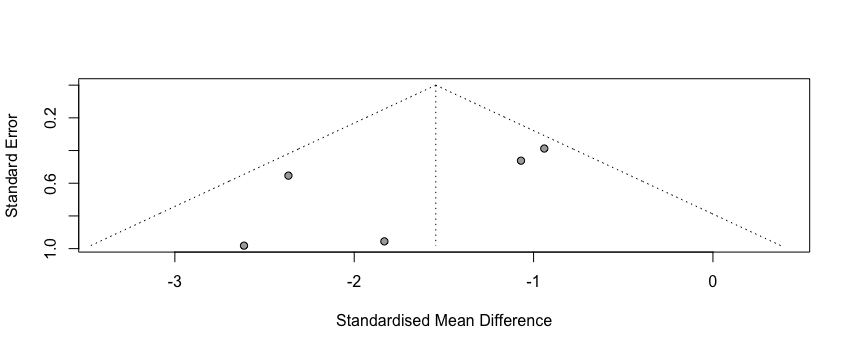


Supplementary material B Figure 3. The funnel plot of the reduction of QMG score


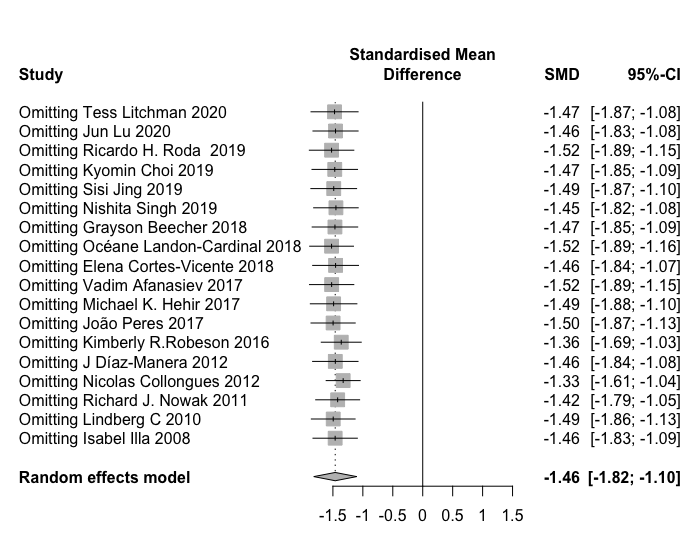


Supplementary material B Figure 4. Sensitivity analysis of the reduction of GC doses


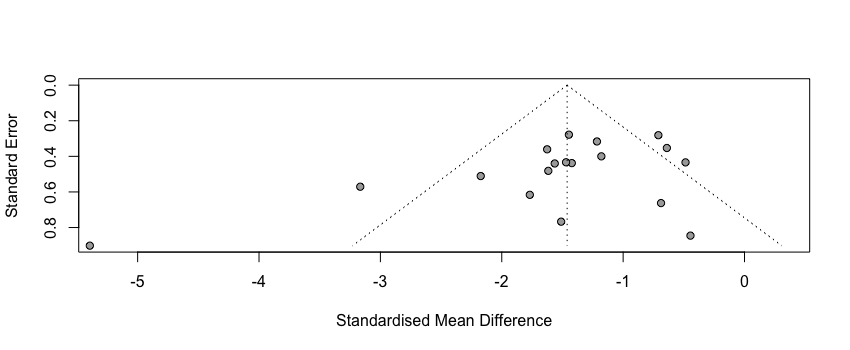


Supplementary material B Figure 5. The funnel plot of the reduction of GC doses


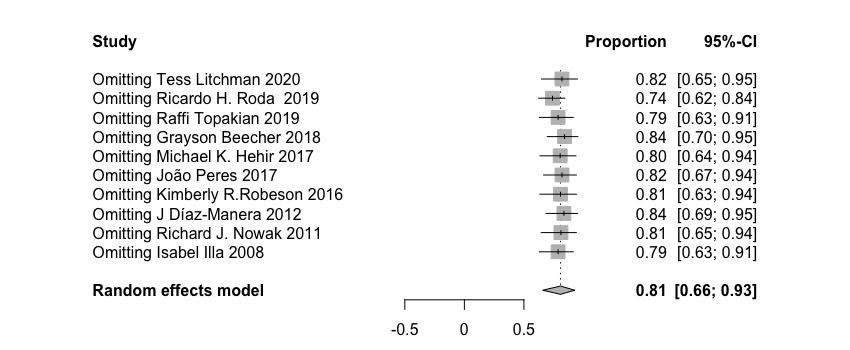


Supplementary material B Figure 6. Sensitivity analysis for the proportion of patients discontinuing oral immunosuppressants


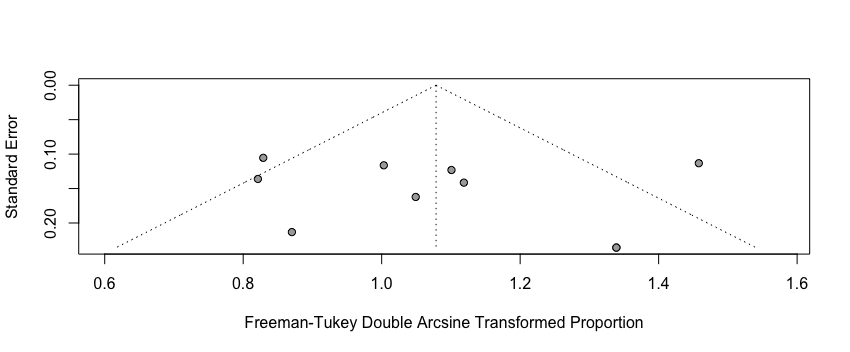


Supplementary material B Figure 7. The funnel plot of the proportion of patients discontinuing oral immunosuppressants
